# A near-infrared-controlled and logic gate sensing platform for the sensitive and specific detection of dual miRNAs

**DOI:** 10.1039/d5ra07697h

**Published:** 2026-02-02

**Authors:** Xinqin He, Mengjie Yang, Xia Zhang, Bin Qiu, Hong Jiang

**Affiliations:** a Department of Obstetrics and Gynecology, The First Affiliated Hospital of Fujian Medical University Fuzhou 350005 China; b Department of Obstetrics and Gynecology, National Regional Medical Center, Binhai Campus of the First Affiliated Hospital, Fujian Medical University Fuzhou 350212 China; c Department of Obstetrics and Gynecology, The First Affiliated Hospital of Xiamen University, School of Medicine, Xiamen University Xiamen 361021 China; d College of Chemistry, Fuzhou University Fuzhou 350108 China summer328cn@163.com; e Department of Reproductive Center, The First Affiliated Hospital of Fujian Medical University Fuzhou 350005 China drjiang2025@163.com

## Abstract

Early detection of endometrial cancer remains challenging because of the lack of sensitive and specific diagnostic methods. This study develops a near-infrared (NIR)-activatable sensing platform with an AND logic gate to simultaneously detect miR-21 and miR-155, two promising endometrial cancer biomarkers. The sensing platform integrates acid-sensitive zeolitic imidazolate framework-8 (ZIF-8)-coated upconversion nanoparticles (UCNP@ZIF-8) with ternary DNA strands, consisting of a BHQ2-modified long strand (S2), a Cy5-labelled short strand (S3), and a photocleavable switch-bearing strand (S1). In a weakly acidic environment, the ZIF-8 shell decomposes to release UCNPs and DNA strands. Under 808 nm excitation, the UCNPs convert NIR light to ultraviolet or visible emission, cleaving the photocleavable linkers on S1 and exposing the toehold domain. The target miRNAs subsequently initiate a toehold-mediated strand displacement reaction, simultaneously displacing S1 and S3, thereby restoring Cy5 fluorescence. The biosensor demonstrated excellent sensitivity, with detection limits of 0.28 nM for miR-21 and 0.35 nM for miR-155. Validation in serum samples revealed recovery rates of 92–103.08%, confirming its potential for clinical application in complex biological environments.

## Abstract 2

Early detection of endometrial cancer remains challenging because of the lack of sensitive and specific diagnostic methods.

## Graphical abstract

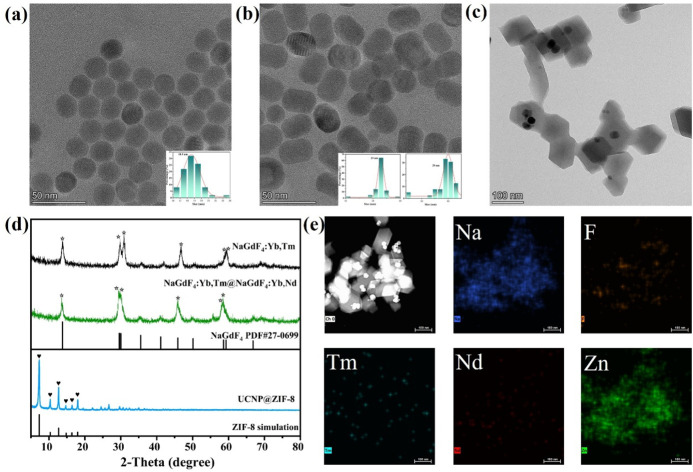

## 1 Introduction

Endometrial cancer is a prevalent gynecologic malignancy with a high mortality rate, posing a serious threat to women's health.^1,2^ Owing to the insidious early symptoms and rapid progression of endometrial cancer, some patients are diagnosed in the middle to late stages and have a poor prognosis.^3^ Therefore, exploring effective methods for predicting therapeutic efficacy is highly important for timely adjustment of treatment plans and improvement of patient prognosis. With the advancement of tumor diagnostic technology, the value of serum biomarkers in efficacy monitoring and prognosis evaluation has become increasingly prominent.^4,5^ MicroRNAs (miRNAs) are endogenous noncoding small molecules that act as key regulators in diverse biological processes,^6^ including cell proliferation, apoptosis, and invasion.^7–9^ Its abnormal expression is closely related to various cancers and is considered a highly promising disease diagnostic biomarker.^10^ Multiple miRNAs have utility as biomarkers for the diagnosis and prognosis of endometrial cancer. For example, miR-21 and miR-155 are abnormally expressed in the serum and tissues of endometrial cancer patients.^11,12^ The simultaneous detection of both miRNAs is expected to offer valuable insights for the early diagnosis and effective monitoring of endometrial cancer.

At present, the commonly used methods for detecting miRNAs include northern blotting,^13^ the microarray method,^14^ and real-time quantitative polymerase chain reaction.^15^ These methods generally have disadvantages, such as low flux, cumbersome operation, and susceptibility to environmental influences. Recent advancements in biosensing technology have driven the establishment of diverse novel miRNA detection approaches, including electrochemiluminescence sensors,^16^ photoelectrochemical sensors,^17^ and fluorescent biosensors.^18^ Owing to their rapid response, good repeatability, and simple equipment, fluorescent biosensors have attracted much attention.^19–21^ However, traditional fluorescence sensors often use organic dyes^22^ or quantum dots^23^ as readout signals, which face limitations, including poor photostability and susceptibility to photobleaching. Furthermore, its excitation source typically lies in the ultraviolet-visible range, which carries high energy and can cause photodamage to biological samples, limiting its application in biosensing. Therefore, it is crucial to develop novel fluorescent probes to achieve accurate and sensitive detection of miRNAs in biological samples.

Lanthanide-doped upconversion nanoparticles (UCNPs) have garnered considerable interest because of their distinctive optical properties.^24^ Unlike traditional luminescent materials, such as quantum dots and organic dyes, UCNPs are capable of absorbing multiple low-energy photons upon exposure to near-infrared (NIR) light (*e.g.*, 808 nm) through the anti-Stokes luminescence process, emitting shorter wavelengths of ultraviolet or visible light.^25,26^ The large spectral separation between excitation and emission effectively minimizes excitation leakage and scattering interference in the detection channel, resulting in an improved signal-to-noise ratio.^27^ Moreover, because NIR excitation lies within the optical transparency window of biological tissues, it experiences reduced tissue absorption and scattering, enabling more efficient excitation in complex biological matrices and substantially suppressing autofluorescence background, which collectively enhances detection sensitivity and reproducibility.^28–30^ In addition, relative to the commonly used 980 nm excitation, 808 nm excitation typically results in lower water absorption and reduced photothermal effects, thereby mitigating the increase in local temperature and potential thermal damage and making it more suitable for long-term, stable excitation and signal acquisition in biological samples.^31^ In addition, UCNPs have the advantages of high luminescence stability^32^ and strong resistance to photobleaching,^33^ which enables them to effectively minimize signal drift and attenuation during prolonged acquisition, thereby improving quantitative accuracy and reproducibility; moreover, they can maintain stable emission under continuous illumination, extend the observation window and enhance the reliability of long-term detection. Therefore, UCNPs can serve as excellent energy donors for constructing biosensors on the basis of luminescence resonance energy transfer (LRET) and have been applied for the detection of miRNAs.^34^ However, existing research has focused mostly on the detection of single miRNA targets. Compared with a single biomarker, the combined detection of multiple biomarkers can more comprehensively reflect the pathological and physiological status of diseases,^35^ which helps to improve the sensitivity and specificity of diagnosis.^36^ Therefore, there is an urgent need to develop new methods that can achieve sensitive and specific detection of multitarget miRNAs.

**Scheme 1:**
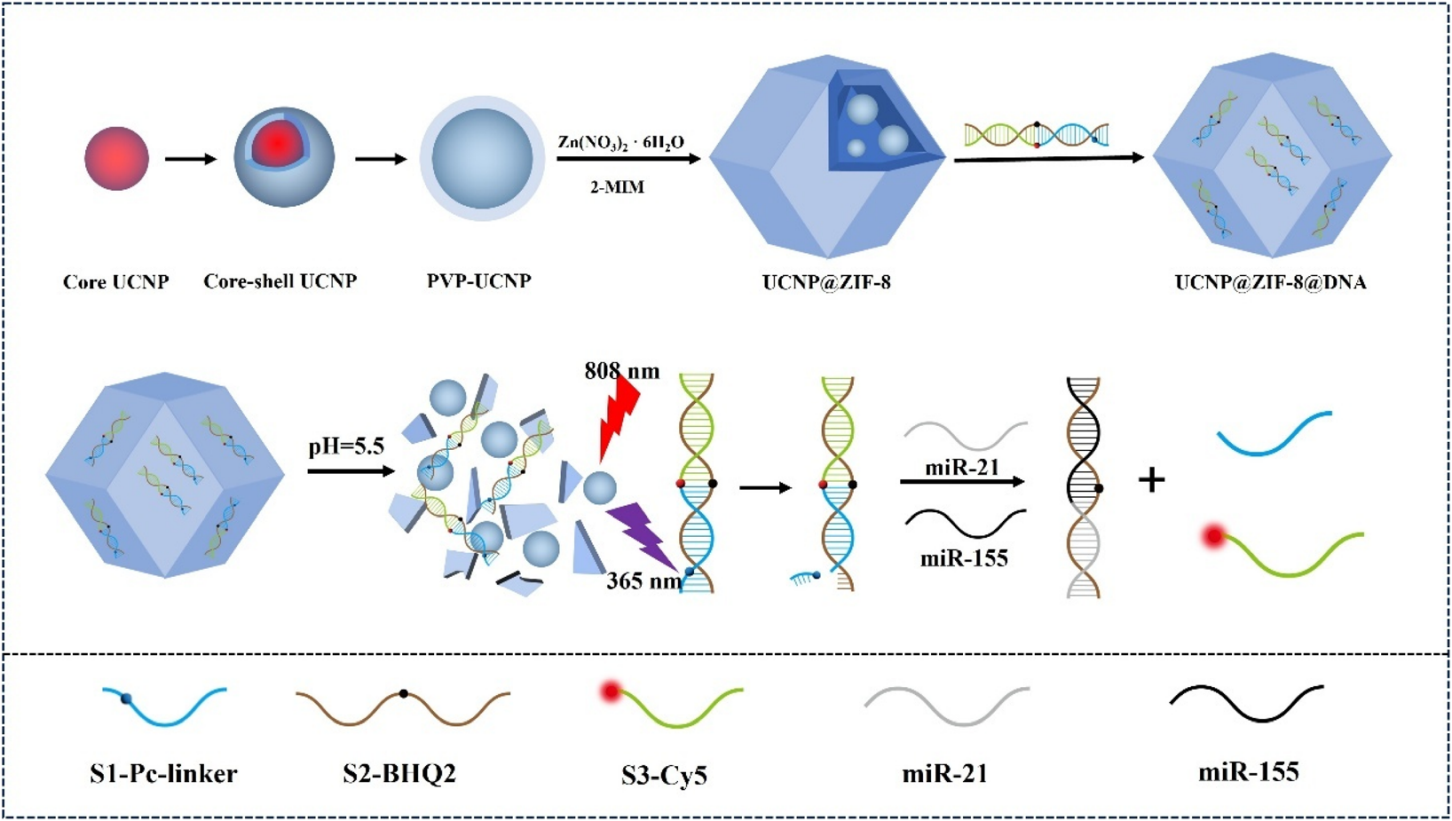
Sensing platform based on the AND logic gate response for dual miRNA measurement.

In this work, a sensing platform that operates on an AND logic gate responsive to miRNA biomarkers was constructed, which precisely regulates the limited LRET process through acid response and light control strategies to achieve sensitivity and specificity detection of the dual tumor markers miR-21 and miR-155 (Scheme 1). The platform is centered around the UCNPs and is coated with a zeolitic imidazolate framework-8 (ZIF-8) layer, which is used to load long DNA strands (S2) containing the quenching group BHQ2, short DNA strands (S1) containing photo cleavage sites (PC linkers), and short DNA strands (S3) carrying Cy5 fluorescent groups. Under weakly acidic conditions, the ZIF-8 shell is cleaved to release DNA strands and UCNPs. Under 808 nm excitation, the UCNPs emit blue light (∼365 nm), selectively cleaving the photocleaving PC linker on S1 and exposing the toehold promoter sequence. The target miRNA undergoes a strand displacement reaction mediated by a toehold, simultaneously replacing S1 and S3, causing Cy5 to move away from the BHQ2 quenching group and fluorescence recovery, thus achieving quantitative detection. This sensing platform combines the AND logic of “acid trigger + light trigger” and the “dual strand collaborative recognition” mechanism, significantly improving the triggering threshold, reducing the risk of background signals and false positives, and has the advantages of easy operation, controllable time and space, and good biocompatibility. It can achieve highly sensitive and specific detection of dual miRNAs in complex biological samples, providing a new strategy for early screening and progression monitoring of endometrial cancer.

## 2 Experimental

### 2.1 Instruments and reagents

The morphology and structure of the samples were characterized *via* high-resolution transmission electron microscopy (HRTEM, Talos F200i). X-ray diffraction (XRD) patterns were recorded on a Rigaku MiniFlex 600 diffractometer, with complementary chemical analysis provided by X-ray photoelectron spectroscopy (XPS). Fourier-transform infrared (FT-IR) spectra were recorded on a Nicolet 5700 spectrometer. The concentrations of metal ions in solution were determined *via* inductively coupled plasma-mass spectrometry (ICP-MS, iCAP 7400). Upconversion emission spectra were recorded on a FluoroMax-4 spectrofluorometer. Zeta potentials were determined *via* a Zetasizer Nano-ZS90 analyzer.

YCl_3_·6H_2_O (99.9%), GdCl_3_·6H_2_O (99.9%), TmCl_3_·6H_2_O (99.9%), NdCl_3_·6H_2_O (99.9%), oleic acid (OA) (95%), 1-octadecene (ODE) (90%), NaOH (A. R.), NH_4_F (98%), 2-methylimidazole (2-MIM) (98%), poly(vinylpyrrolidone) (PVP) (K30), *N*,*N*-dimethylformamide (DMF) (A. R.), and zinc nitrate hexahydrate [Zn(NO_3_)_2_·6H_2_O] (99%) were purchased from Aladdin (Shanghai, China). Anhydrous ethanol (A. R.), methanol (A. R.), and cyclohexane (A. R.) were purchased from Sinopharm Chemical Reagent Co., Ltd (Shanghai, China). All nucleic acid sequences were purchased from Sangon Biotech Co., Ltd (Shanghai). The DNA and RNA strands were as follows:

S1: 5′-TAGCTTA/iPCLink/TCAGACTGATGTTGATTAATGC-3′

S2: 5′-TTTTAATACCCCTATCACGAT/iBHQ2dT/AGCATTAATCAACATCAGTCTGATAAGCTA-3′

S3: 5′-Cy5-TAATCGTGATAGGGGTATTCGAT-3′

miR-21: 5′-UAGCUUAUCAGACUGAUGUUGA-3′

miR-155: 5′-UUAAUGCUAAUCGUGAUAGGGGU-3′

miR-222: 5′-AGCUACAUCUGGCUACUGGGU-3′

miR-122: 5′-UGGAGUGUGACAAUGGUGUUUG-3′

### 2.2 Synthesis of nanoparticles

#### 2.2.1 Synthesis of NaGdF_4_:Yb,Tm

Hydrated rare-earth chlorides, including GdCl_3_·6H_2_O (0.79 mmol), YbCl_3_·6H_2_O (0.20 mmol), and TmCl_3_·6H_2_O (0.01 mmol), were placed in a 100 mL three-neck round-bottom flask along with a mixture of OA (6 mL) and ODE (15 mL). Under argon protection, the mixture was heated to 160 °C and held at this temperature for 30 min until a clear solution formed, indicating complete dissolution, before being cooled to 40 °C. To initiate nucleation, a methanolic solution (10 mL) containing NH_4_F (0.148 g) and NaOH (0.10 g) was introduced, and the resulting suspension was then held at 40 °C for 30 min. The temperature was then raised to 110 °C and held for 20 min to remove residual water and methanol, followed by heating to 300 °C for 1 h to produce the nanocrystals. The resulting product was harvested by centrifugation, subjected to sequential washing with ultrapure water and absolute ethanol (three times each), and ultimately redispersed in 5.0 mL of cyclohexane for storage as oleat-capped NaGdF_4_:Yb,Tm core nanoparticles.

#### 2.2.2 Synthesis of NaGdF_4_:Yb,Tm@NaGdF_4_:Yb,Nd

Hydrated rare-earth chlorides, including GdCl_3_·6H_2_O (0.50 mmol), YbCl_3_·6H_2_O (0.10 mmol), and NdCl_3_·6H_2_O (0.40 mmol), were combined in a 250 mL three-neck round-bottom flask along with a mixture of OA (10.5 mL) and ODE (10.5 mL). Under an argon atmosphere, the mixture was heated to 160 °C and maintained at that temperature for 30 min until complete dissolution of the salts was achieved, after which it was cooled to 40 °C. A cyclohexane dispersion (5 mL) of the as-prepared bare-core UCNPs from Section 2.2.1 was introduced and stirred for 30 min. The mixture was then heated to 80 °C and maintained for 30 min to remove cyclohexane, followed by cooling to room temperature. Subsequently, a methanolic solution (10 mL) of NH_4_F (0.148 g) and NaOH (0.10 g) was introduced, and the resulting suspension was incubated at 40 °C for 30 min to promote shell growth. The temperature was first ramped to 110 °C and held for 20 min to remove residual water and methanol, then subsequently raised to 300 °C and maintained for 1 h to facilitate the formation of core–shell nanocrystals.

### 2.3 Synthesis of UCNP@ZIF-8 nanomaterials

The surface ligands on the UCNP cores were first removed. Specifically, 2.5 mL of the as-prepared core–shell UCNP dispersion was added to ethanol (15 mL) containing concentrated HCl (150 µL) and sonicated for 1 h. The nanoparticles were collected by centrifugation at 10 000 rpm for 10 min, and the precipitate was subjected to three washing cycles with an ethanol/water mixture, yielding ligand-free UCNPs. The ligand-free UCNPs were then redispersed in ethanol (15 mL) containing PVP (0.2 g) and subjected to stirring at room temperature for 24 h. The product was collected by centrifugation at 10 000 rpm for 10 min and then washed three times with absolute ethanol, and finally dispersed in methanol (5 mL) for subsequent use.

A seed-mediated strategy using UCNPs-PVP was employed to grow ZIF-8 shells. Briefly, 500 µL of the UCNP-PVP dispersion was added to 4 mL of a methanolic 2-MIM solution (0.82 g). A methanolic solution of Zn(NO_3_)_2_·6H_2_O (5 mL, 0.298 g) was then added dropwise to the above mixture, followed by continued stirring at room temperature for 24 h. The resulting mixture was centrifuged and washed with methanol to remove excess ligands, and the resulting product was redispersed in methanol (5 mL).

### 2.4 Construction of UCNP@ZIF-8@DNA sensors

To construct a DNA logic gate for dual-miRNA (miR-155 and miR-21) measurement, S1, S2, and S3 (5 µL each, 10 µM) were combined in HEPES buffer (125 mM HEPES, 685 mM NaCl, pH 7.4). The mixture was heated at 95 °C for 10 min, followed by gradual cooling to 25 °C to form the duplex DNA logic gate. Next, the DNA duplex (5 µL, 10 µM) was first mixed with the prepared UCNP@ZIF-8 dispersion (50 µL) and then 100 µL of HEPES buffer (125 mM HEPES, 685 mM NaCl, pH 7.4) was added. After incubation for 1 h and washing by centrifugation, UCNP@ZIF-8@DNA was obtained.

### 2.5 Fluorescence determination of miR-21 and miR-155

An aliquot of UCNP@ZIF-8@DNA (30 µL) was mixed with PBS (170 µL, pH 5.4). Then, a PBS solution containing the target miRNA at different concentrations was added. For the miR-21 assays, varying concentrations of miR-21 were introduced into the UCNP@ZIF-8@DNA/PBS mixture in the presence of an excess of miR-155. For the miR-155 assays, varying concentrations of miR-155 were added in the presence of an excess of miR-21. The mixtures were subjected to a 30-min incubation at 37 °C, irradiated with 808 nm near-infrared light for 40 min to trigger strand-displacement, and then maintained at 37 °C for an additional 2 h. Fluorescence emission spectra were monitored *via* an excitation wavelength of 643 nm and collected in the 660–700 nm range.

### 2.6 Fluorescence dual miRNAs in real samples

Before miRNA analysis, the samples were diluted 1 : 20 with PBS (pH 7.4). Quantification was performed *via* the standard-addition method following the same procedure as in Section 2.5. Recoveries were evaluated by spiking the assay system with graded concentrations of the target miRNAs.

## 3 Results and discussion

### 3.1 Characterization of the synthesized nanomaterials

**Fig. 1:**
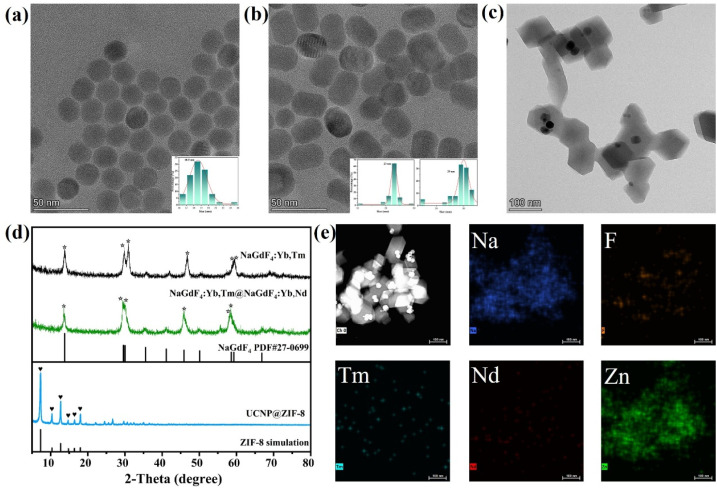
(a) TEM image of the core UCNPs (NaGdF_4_:Yb,Tm); (b) TEM images of core–shell UCNPs (NaGdF_4_:Yb,Tm@NaGdF_4_:Yb,Nd); (c) TEM images of the UCNP@ZIF-8 composite; (d) XRD patterns of the nanomaterials at various stages; (e) element surface distribution diagram of the UCNP@ZIF-8 composite.

The morphology, structure, and chemical composition of the sensing probe material were characterized *via* TEM, XRD, and EDS. As presented in Fig. 1a, the core UCNPs (NaGdF_4_:Yb,Tm) exhibit a regular spherical morphology with a uniform size distribution and a mean diameter of approximately 18.5 nm. Fig. 1b shows that after the shell layer (NaGdF_4_:Yb,Nd) is coated *via* epitaxial growth, the morphology changes from spherical to ellipsoidal, with short- and long-axis sizes of approximately 23 nm and 29 nm, respectively, indicating uniform shell growth and a controllable structure. Fig. 1c further shows the successful growth of ZIF-8 on the core–shell UCNPs surface, the composite exhibited a typical ZIF-8 dodecahedron morphology, and the ellipsoidal core–shell UCNPs encapsulated inside the material could be clearly identified, thus confirming that ZIF-8 was successfully encapsulated on the surface of the UCNPs.

The XRD pattern in Fig. 1d further confirms the crystalline structure of the synthesized material. The diffraction peaks of both the core and core–shell UCNPs, located at 2*θ* ≈ 17.005°, 29.655°, 30.002°, 42.717°, 52.880°, and 53.446°, match well with the reference pattern for hexagonal-phase NaGdF_4_, indicating high crystallinity of the samples. In the XRD profile of the UCNP@ZIF-8 composite, in addition to the retained UCNP diffraction features, characteristic peaks are observed at 2*θ* ≈ 7.30°, 10.35°, 12.70°, 14.80°, 16.40°, and 18.00°, which is consistent with the simulated pattern of ZIF-8. These results confirm the successful formation of a crystalline ZIF-8 framework and further verify the effective encapsulation of the UCNPs within the ZIF-8 matrix. Moreover, the EDS mapping in Fig. 1e shows that key elements such as Na, F, Tm, Nd, and Zn are uniformly distributed in the composite. Collectively, these characterization data confirm the successful fabrication of core UCNPs, core–shell UCNPs, and the UCNP@ZIF-8 composite.

**Fig. 2:**
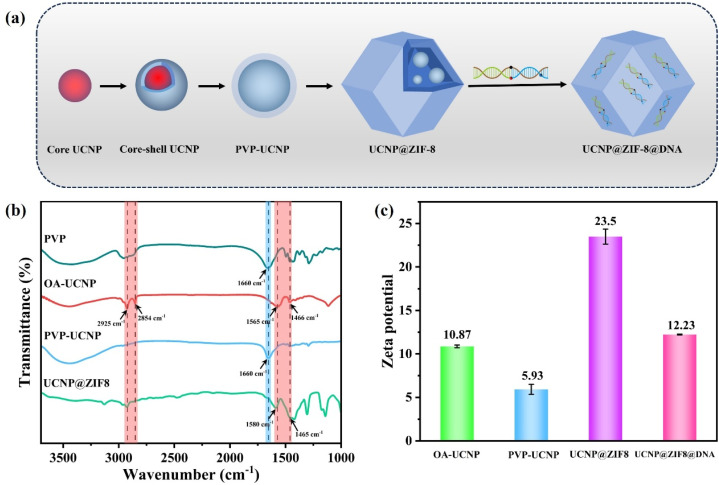
(a) Schematic diagram of the synthesis of the UCNP@ZIF-8@DNA composite; (b) FTIR spectra of the nanomaterials at different stages; (c) zeta potential of the nanomaterials at different stages.

The molecular structure, surface functional groups, and surface potential of the material were subsequently characterized *via* FT-IR and zeta potential. Fig. 2a shows the step-by-step preparation process of the nanomaterials. As presented in the FTIR spectrum (Fig. 2b), the OA-modified UCNPs exhibit characteristic peaks at 2925 and 2854 cm^−1^ that are assigned to the asymmetric and symmetric stretching vibrations of the –CH_2_– alkyl chains, respectively. Meanwhile, those at 1565 and 1466 cm^−1^ correspond to the stretching vibrations of the carboxylate group (–COO^−^). After modification with PVP, the spectrum showed a strong peak at 1660 cm^−1^, corresponding to the C=O stretch, indicating that PVP had successfully coated the surface of the UCNPs. In the FTIR spectrum of the UCNP@ZIF-8 composite, the peak at 1580 cm^−1^ is characteristic of the C=N stretch on the imidazole ring. Conversely, the absorption at 1465 cm^−1^ can be assigned to the in-plane antisymmetric and symmetric C–H bending vibrations of the 2-methylimidazole ring.

The zeta potential results (Fig. 2c) show that the surface potential of the OA-modified UCNPs is approximately +10.87 mV. After modification with the nonionic surfactant PVP, the surface potential of the material tends to be neutral at approximately +5.93 mV. After coating with ZIF-8, the surface potential significantly increased to approximately +23.5 mV because of the contribution of uncoordinated Zn^2+^ and imidazole ring nitrogen atoms in the outer layer. Finally, when negatively charged DNA (*via* its phosphate backbone) adsorbs onto the UCNP@ZIF-8 surface, it partially neutralizes its positive charge, resulting in a decrease in surface potential to approximately +12.23 mV. In summary, the FTIR spectrum and the trend of the zeta potential confirmed the gradual transformation process from OA-UCNPs to PVP-UCNPs, then to UCNP@ZIF-8, and finally to UCNP@ZIF-8@DNA, as well as the successful transformation of the chemical properties of the material surface at each stage.

**Fig. 3:**
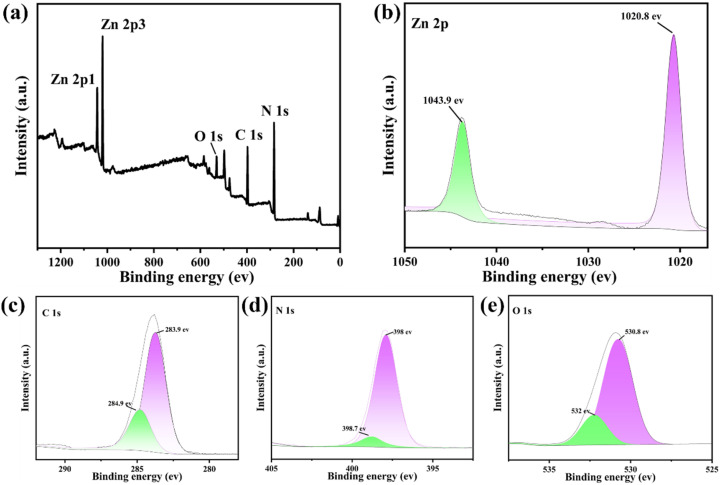
XPS spectra of the UCNP@ZIF-8 composite: (a) full spectrum; (b) Zn 2p peak; (c) C 1s peak; (d) N 1s peak; (e) O 1s peak.

To further confirm the chemical composition and elemental valence states, the UCNP@ZIF-8 composite was subjected to XPS analysis. As shown in Fig. 3a, full XPS spectrum displays distinct peaks for C 1s, N 1s, O 1s, and Zn 2p, whereas no other metal impurity peaks are detected, indicating that the outer layer of the sample is mainly composed of a ZIF-8 organic skeleton with Zn–N coordination. In Fig. 3b, the high-resolution spectrum of Zn 2p shows two characteristic peaks with binding energies located at 1020.8 eV (Zn 2p_3/2_) and 1043.9 eV (Zn 2p_1/2_), which are consistent with the electronic state of Zn^2+^. In Fig. 3c, the C 1s spectrum can be fitted with two peaks at 283.9 eV (C–C) and 284.9 eV (C–N/C=N from the imidazole ring and organic framework). In Fig. 3d, the binding energy of N 1s is mainly concentrated in the range of 398.0–399.0 eV, corresponding to the imidazole ring nitrogen atom coordinated with Zn^2+^, which further confirms the formation of Zn–N coordination bonds. The O 1s spectrum in Fig. 3e exhibits two peaks at 530.8 eV and 532.0 eV; the former is attributed to the possible presence of trace surface Zn–O species, whereas the latter is typically associated with C–O single bonds. The above XPS analysis results are consistent with the previous morphology and structural characterization, fully confirming that the UCNPs were successfully encapsulated within the ZIF-8 skeleton and that the chemical valence states of each element in the composite material are consistent with the theoretical expectations.

### 3.2 Cracking behavior of UCNP@ZIF-8 under acidic conditions

**Fig. 4:**
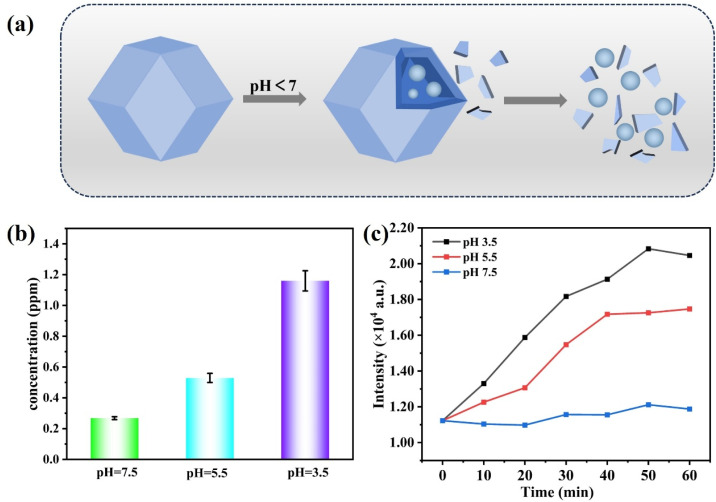
(a) Schematic diagram of acid-responsive cracking of the UCNP@ZIF-8 composite; (b) the concentration of Zn^2+^ in different pH solutions; (c) the fluorescence recovery curves of UCNP at different pH values.

To effectively trigger strand displacement reactions in the acidic microenvironment of tumors, an acid-responsive composite nanocarrier based on the UCNP@ZIF-8 composite was constructed. As shown in Fig. 4a, at pH < 7, the ZIF-8 shell gradually degrades, releasing the encapsulated UCNPs and DNA strands. To evaluate the acid dissociation behavior, the UCNP@ZIF-8 composite was incubated for 60 min under different pH conditions (3.5–7.5), and the release of Zn^2+^ was detected *via* ICP-MS. Fig. 4b shows that as the pH decreases, the concentration of Zn^2+^ in the solution gradually increases, indicating that the degree of ZIF-8 shell dissociation and cracking significantly increases with increasing acidity. The fragmentation process of the UCNP@ZIF-8 composite can also be monitored by changes in the fluorescence of UCNP. As shown in Fig. 4c, at pH 7.5, the fluorescence intensity of the UCNPs showed little variation over time, indicating that the ZIF-8 structure was intact and effectively encapsulated and quenched the fluorescence of the UCNPs. However, when the acidity increased, the fluorescence intensity of UCNP steadily increased, indicating that the carrier began to crack gradually and release UCNP. In summary, the UCNP@ZIF-8 composite can effectively degrade and release UCNPs and DNA strands in acidic environments, providing a necessary foundation for triggering strand displacement reactions in the tumor microenvironment in the future.

### 3.3 The detection principle and feasibility of the proposed method

**Fig. 5:**
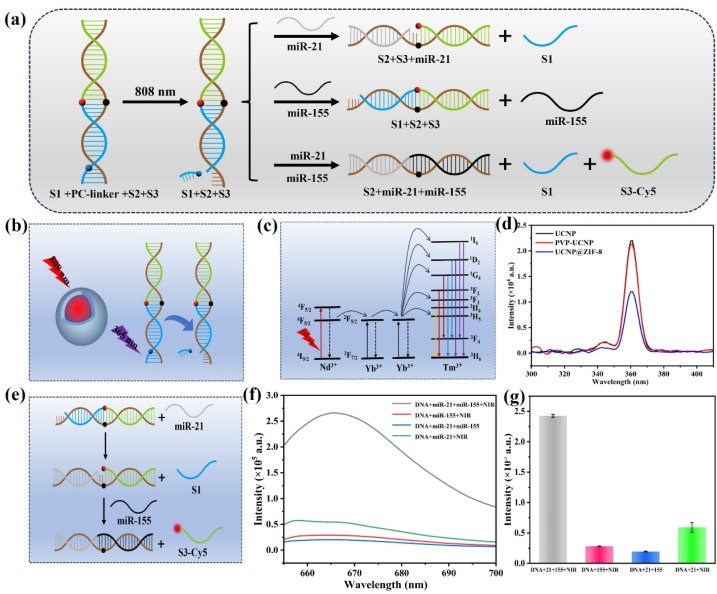
(a) Schematic diagram of AND logic gate operation based on NIR light excitation, with miR-21 and miR-155 as input signals; (b) schematic diagram of the process of light-controlled release and recognition site exposure; (c) energy transition pathways of rare earth ions in UCNPs; (d) comparison of fluorescence spectra of different nanomaterials under 365 nm excitation; (e) schematic diagram of the mechanism of the strand displacement reaction-induced fluorescence recovery of Cy5; (f and g) fluorescence response spectra of the detection system at 665 nm under different reaction conditions.

This study developed an NIR-activatable sensor for the sensitive and specific determination of miR-21 and miR-155. As illustrated in Fig. 5a, the sensor employs a DNA-based AND logic gate that ensures a response only when both miR-21 and miR-155 are present. The gate structure consists of a DNA duplex that is assembled by complementary base pairing of three single strands, S1, S2, and S3. Under 808 nm irradiation, the UCNPs are capable of converting NIR light into ultraviolet-visible emission, which cleaves the PC linkers on the S1 strand and selectively exposes the recognition sites (Fig. 5b). The exposed region is then recognized and bound by miR-21, initiating a strand displacement reaction. Subsequently, miR-155 displaces the S3 strand through competitive binding, leading to recovery of the Cy5 fluorescence signal (Fig. 5e).

To achieve the aforementioned optical control process, we synthesized Yb^3+^, Tm^3+^ and Nd^3+^-doped UCNPs and investigated their energy transfer pathways. As shown in Fig. 5c, under 808 nm excitation, Nd^3+^ absorbs photon energy and transfers it to Yb^3+^*via* nonradiative relaxation. Yb^3+^ then relays the energy stepwise to Tm^3+^, which achieves upconversion luminescence through multistep excited-state absorption, producing several emission bands. Notably, ultraviolet emission at approximately 365 nm is particularly prominent. Fig. 5d displays the fluorescence spectrum of the synthesized UCNPs at 365 nm, confirming their ability to efficiently cleave the PC linkers and thereby activate subsequent strand displacement reactions. Furthermore, coating the UCNPs with a ZIF-8 shell resulted in a measurable decrease in fluorescence intensity, verifying the successful preparation of the UCNP@ZIF-8 composite structure.

The feasibility of the proposed method, which is based on an AND logic gate and an NIR light response, was also studied. Fig. 5(f and g) shows the fluorescence spectra of the detection system under various experimental parameters. With the coexistence of DNA, miR-21, and miR-155 and the application of NIR light, significant recovery of the Cy5 fluorescence signal can be observed. However, when only a single miRNA (such as DNA + miR-155 + NIR or DNA + miR-21 + NIR) was added or NIR light was not applied (DNA + miR-21 + miR-155), the strand displacement reaction could not be effectively completed, and the Cy5 fluorescence signal remained at an extremely weak level. The above results confirm that the proposed NIR-activatable sensor for the determination of miR-21 and miR-155 is feasible.

### 3.4 Optimization of detection conditions

**Fig. 6:**
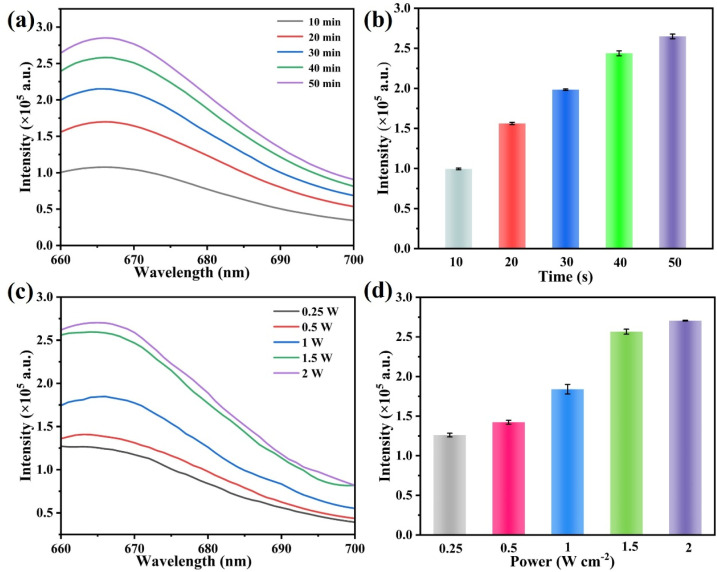
(a and b) Effects of different illumination times and (c and d) different illumination powers on the fluorescence intensity of the system at 665 nm.

Before the detection of the target miRNAs, two key parameters in the strand displacement reaction, illumination time and illumination power, were optimized. The irradiation time of NIR light directly influences the cleavage efficacy of PC linkers on the S1 strand, thereby regulating the intensity of the Cy5 fluorescence signal. First, the changes in Cy5 fluorescence intensity were investigated under different illumination times (10–50 min). Fig. 6(a and b) exhibits that as the illumination time increased, the fluorescence signal continued to increase, and 40 min was ultimately selected as the optimal illumination time. The effects of light power on fluorescence recovery were further evaluated. As presented in Fig. 6(c and d), under the same irradiation time, the Cy5 fluorescence intensity exhibited a steady rise with increasing laser power, and the signal tended to stabilize when the power reached 1.5 W cm^−2^. Therefore, 1.5 W cm^−2^ was determined as the optimal illumination power and was applied to all subsequent experiments.

### 3.5 Evaluation of detection performance

**Fig. 7:**
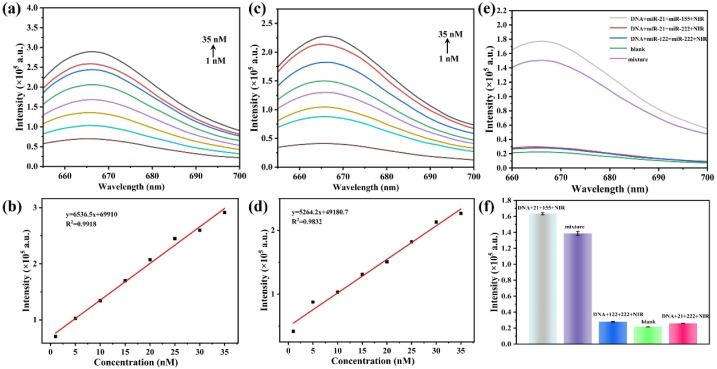
(a) Fluorescence response curves at different concentrations of miR-21; (b) linear relationship between the fluorescence intensity and the concentration of miR-21; (c) fluorescence response curves at different concentrations of miR-155; (d) linear calibration curve of fluorescence intensity *versus* miR-155 concentration; (e and f) specificity assessment of the sensor towards miR-21 and miR-155.

To assess the detection performance of the developed sensor, the response of the sensing system to dual miRNAs (miR-21 and miR-155) was measured under the aforementioned optimized conditions. As shown in Fig. 7a, the fluorescence intensity of the sensing system at 665 nm gradually increased with increasing miR-21 concentration. Fig. 7b further indicates that there is a good linear relationship between the fluorescence intensity and the miR-21 concentration across 1–35 nM (*R*^2^ = 0.9918). The linear relationship is fitted to the equation *y* = 6536.5*x* + 69 910 (where *y* is the fluorescence intensity and *x* is the miR-21 concentration). The limit of detection (LOD), calculated *via* a signal-to-noise ratio (S/N) of 3, is determined to be 0.28 nM. Similarly, the sensing system exhibited concentration-dependent fluorescence enhancement behavior toward miR-155 (Fig. 7c). A linear relationship (*R*^2^ = 0.9832) existed between the fluorescence intensity and the miR-155 concentration across the 1–35 nM range, and the fitting equation was *y* = 5264.2*x* + 49 180.7, with a detection limit of 0.35 nM (Fig. 7d). Compared with several reported methods (Table 1), although the LOD of the proposed method is slightly lower or comparable, it does not require enzyme involvement and signal amplification, and can simultaneously detect two miRNAs, indicating that the designed nanoprobes are suitable for simple and sensitive multi miRNA detection.

**Table 1:** Comparative evaluation of miR-21 and miR-155 detection platforms

Material	Amplification strategy	Target	Linear range (nM)	Detection limit (nM)	Reference
MnO_2_ + DNA	CHA	miR-21	1–50	0.33	37
DNA	APE1/CHA	miR-21	2.5–40	0.22	38
DNA	CHA	miR-21	0.5–20	0.441	39
DNA	CHA	miR-155	0.5–30	0.35	40
DNA-AgNCs	DSN	miR-155	1–600	0.86	41
HeminG + MOF	DNAzyme/HCR	miR-155	0–200	0.17	42
UCNP + DNA	—	miR-21 + miR-155	1–35	0.28	This work
				0.35	

Furthermore, to validate the specificity of the developed assay for the target miRNAs, we investigated the interference of other miRNAs in the detection system. As shown in Fig. 7(e and f), significant recovery of the Cy5 fluorescence signal occurred only in the presence of both miR-21 and miR-155. The single target miRNA or other interfering miRNAs did not cause a significant signal response, indicating that the probe has excellent specific recognition ability and holds considerable promise for detecting targets in complex biological samples.

### 3.6 Detection in actual samples

To evaluate the applicability and reliability of the fabricated NIR-triggered logic gate sensing platform in actual samples, the constructed biosensor was applied to analyze miR-21 and miR-155 in serum samples. Under the established optimal parameters, validation was carried out through spiked recovery experiments. The target miRNAs at three concentrations (1, 5, and 10 nM) were added to the diluted serum samples, and each concentration was measured in parallel three times (*n* = 3). As presented in Table 2, the recovery rates of miR-21 and miR-155 in spiked samples ranged from 92% and to 103.08%, with relative standard deviations (RSD) below 4.55%, indicating that the proposed assay has good accuracy and reproducibility. The above results demonstrate that the sensor can maintain stable performance in complex matrices and can be used for accurate quantitative analysis of miR-21 and miR-155 in actual serum samples.

**Table 2:** Results of miRNA detection in serum samples

Sample	Added (nM)	Found (nM)	Recover (%)	RSD (%)
Serum (miR-21)	1	0.92	92	4.55
	5	5.064	103.08	1.02
	10	10.18	100.69	0.8
Serum (miR-155)	1	0.983	98.74	3.21
	5	4.83	96.78	3.22
	10	10.066	100.66	1.29

## 4 Conclusion

In this work, we developed an NIR-triggered logic gate sensing platform for the simultaneous detection of miR-21 and miR-155. The integration of UCNPs with the ZIF-8 framework enabled precise spatiotemporal control through acid-responsive degradation and NIR-activated strand displacement reactions. Systematic characterization confirmed the successful fabrication of the core–shell nanocomposite with well-defined structural and optical properties. The proposed sensor exhibited excellent sensitivity, with detection limits of 0.28 nM for miR-21 and 0.35 nM for miR-155. When validated in serum samples, the platform delivered recovery rates ranging from 92% to 103.08%, with RSDs below 4.55%, underscoring its reliability and potential for clinical application in complex biological matrices. This approach provides a novel methodology for multiplexed miRNA detection in complex biological samples and offers promising potential for early screening and progression monitoring of endometrial cancer. In future work, we will further validate the platform using clinical samples and expand its adaptability for detecting other cancer-related biomarkers.

## Ethical statement

All experiments were performed in accordance with the Guidelines of The First Affiliated Hospital of Fujian Medical University, and experiments were approved by the ethics committee at The First Affiliated Hospital of Fujian Medical University. Informed consents were obtained from human participants of this study.

## Author contributions

Xinqin He, Bin Qiu and Hong Jiang designed research. Xinqin He, Mengjie Yang and Xia Zhang performed the experiments. Xinqin He and Mengjie Yang analyzed data. All author wrote and revised the manuscript.

## Conflicts of interest

The authors declare that they have no known competing financial interests or personal relationships that could have appeared to influence the work reported in this paper.

## Data Availability

The authors confirm that the data supporting the findings of this study are available within the article.
